# The Impact of Patient Online Access to Computerized Medical Records and Services on Type 2 Diabetes: Systematic Review

**DOI:** 10.2196/jmir.7858

**Published:** 2018-07-06

**Authors:** Freda Mold, Mary Raleigh, Nouf Sahal Alharbi, Simon de Lusignan

**Affiliations:** ^1^ School of Health Sciences Faculty of Health and Medical Sciences University of Surrey Guildford United Kingdom; ^2^ Florence Nightingale Faculty of Nursing, Midwifery & Palliative Care King's College London London United Kingdom; ^3^ Department of Health Sciences College of Applied Studies & Community Service King Saud University Riyadh Saudi Arabia; ^4^ Department of Clinical and Experimental Medicine Faculty of Health and Medical Sciences University of Surrey Guildford United Kingdom

**Keywords:** medical records, online access, online services, medical records systems, computerized, computers, primary care, type 2 diabetes mellitus

## Abstract

**Background:**

Online access to computerized medical records has the potential to improve convenience, satisfaction, and care for patients, and to facilitate more efficient organization and delivery of care.

**Objective:**

The objective of this review is to explore the use and impact of having online access to computerized medical records and services for patients with type 2 diabetes mellitus in primary care.

**Methods:**

Multiple international databases including Medline, Embase, CINAHL, PsycINFO and the Cochrane Library were searched between 2004 and 2016. No limitations were placed on study design, though we applied detailed inclusion and exclusion criteria to each study. Thematic analysis was used to synthesize the evidence. The Mixed Methods Appraisal Toolkit was used to appraise study quality.

**Results:**

A search identified 917 studies, of which 28 were included. Five themes were identified: (1) disparities in uptake by age, gender, ethnicity, educational attainment, and number of comorbidities, with young men in full-time employment using these services most; (2) improved health outcomes: glycemic control was improved, but blood pressure results were mixed; (3) self-management support from improved self-care and shared management occurred especially soon after diagnosis and when complications emerged. There was a generally positive effect on physician-patient relationships; (4) accessibility: patients valued more convenient access when online access to computerized medical records and services work; and (5) technical challenges, barriers to use, and system features that impacted patient and physician use. The Mixed Methods Appraisal Toolkit rated 3 studies as 100%, 19 studies as 75%, 4 studies as 50%, and 1 study scored only 25%.

**Conclusions:**

Patients valued online access to computerized medical records and services, although in its current state of development it may increase disparities. Online access to computerized medical records appears to be safe and is associated with improved glycemic control, but there was a lack of rigorous evidence in terms of positive health outcomes for other complications, such as blood pressure. Patients remain concerned about how these systems work, the rules, and timeliness of using these systems.

## Introduction

Worldwide, in 2015, 415 million adults aged 20 to 79 years were estimated to have diabetes; and this figure is expected to rise to 642 million by 2040 [1]. The most common type of diabetes is type 2 (type 2 diabetes mellitus, T2DM) and the number of T2DM patients in the UK is steadily growing [2]. Currently, there are 3.2 million people with T2DM, and by 2025 this figure is estimated to reach 5 million [3,4]. A further 630,000 people are predicted to have undiagnosed T2DM [5]. The impact of T2DM is considerable, with the expenditure for treating this condition—and its complications—currently costing the National Health Service £8.8 billion a year, which is over 8% of its annual budget. This expenditure is expected to rise to £15.1 billion by 2035 [6].

Online access to medical records has the potential to support patient-centered care, to improve convenience for patients, and to improve patient satisfaction. Empowering patients by giving them greater access to their medical records and to link online services may, not only assist in self-management of their conditions, but also facilitate organization and delivery of care [7,8]. However, use of these technologies by patients is also a burden for health care providers and there are concerns about privacy and confidentiality [9,10]. Progress has been made in the US health system [11,12], with organizations such as Kaiser Permanente accruing 2 million members who signed up for online services such as appointment bookings, viewing of test results, and emails [13]. However, progress in this regard has been more limited elsewhere in the world.

National systems provide online patient portals separate from their health providers computerized medical records (CMRs) have not been successful in both France and the UK. The French system, Dossier Medical Personnel, was established in 2004 and is a secure CMR system enabling patients direct access to their personal health records. However, by 2013 only 0.31% of the population had opened an account [14]. The English system, “HealthSpace” [15,16], had similarly limited successes with only 0.13% (2913 of the invited 2,442,215) actually signing up and activating their advanced account [16]. Additionally, health professionals in the UK also remain concerned about security, privacy [17-21], and legal constraints [22] of such systems.

In the UK, policy has changed to one which promotes patient access to their medical records via their primary care provider’s CMR system [23]. This access also includes patient online services such as booking appointments, viewing test results, and ordering of prescription refills (repeat prescriptions) [24]. However, email access, which is often part of the provision of such services, is not currently planned.

The aim of this review is to explore the use and impact of having online access to CMR and services for patients with T2DM in primary care.

The objectives are:

- To identify users and nonusers of patient online access to CMRs and services for adults with T2DM (and their caregivers).
- To identify the impact of patients having online access to their CMRs and services in relation to T2DM health outcomes.
- To describe how patient online access to CMRs and services impacts disease management, health delivery, and service access for patients with T2DM.
- To identify any technical challenges, barriers to use and system features which may impact on patients’ uptake and use of online access to CMRs and services.

In identifying these factors, we intend to enhance knowledge of who, why (for what reasons), and when patients use or do not use online access to CMRs and services to manage their diabetes. This is important if we are to identify potential gaps in new service delivery methods; and critical if we are to design innovative services that bridge gaps in current care and design services which are accessible to all.

## Methods

### Review Structure

**Figure 1:**
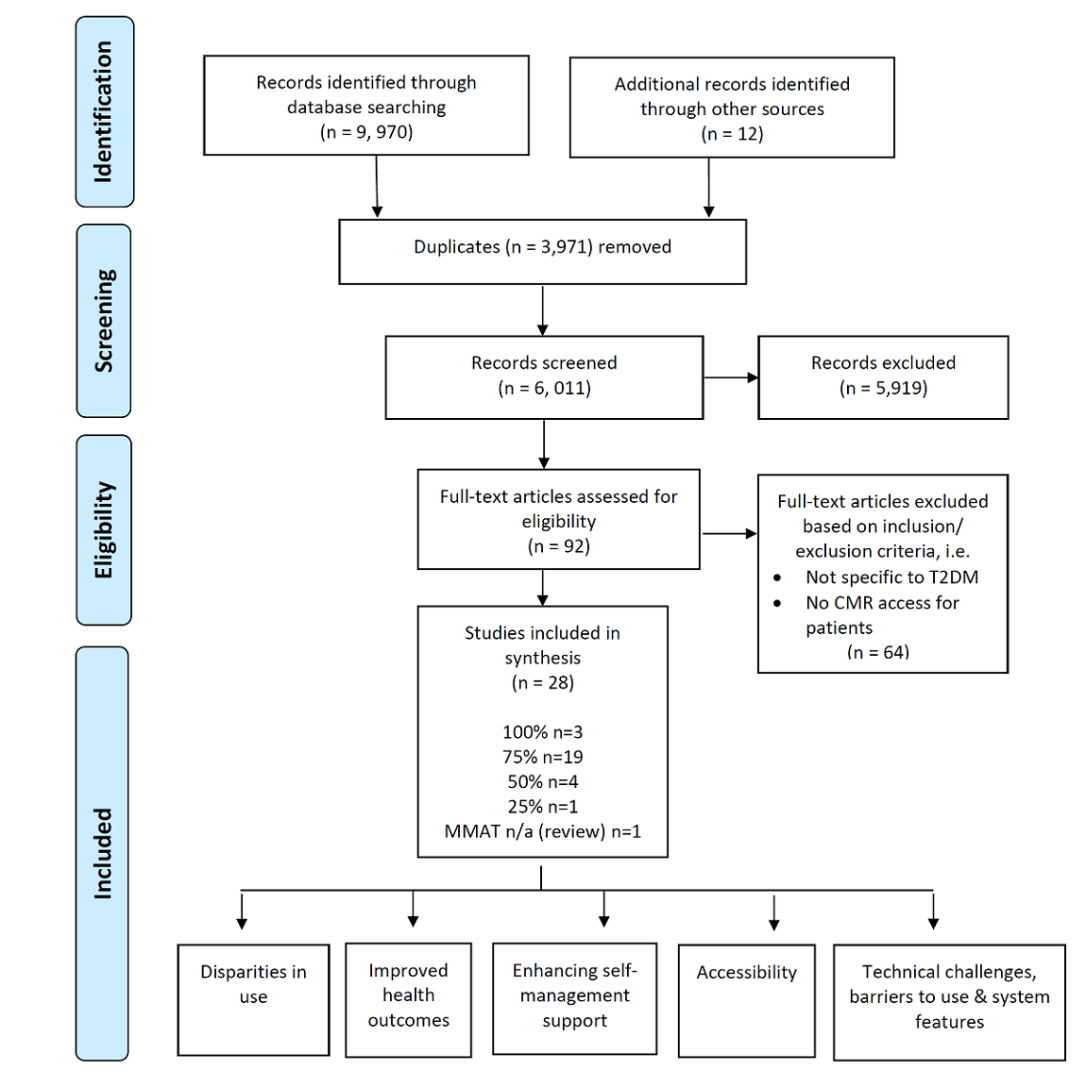
Preferred Reporting Items for Systematic Reviews and Meta-Analysis (PRISMA) flow diagram used for the systematic review. CMR: computerized medical record, MMAT: Mixed Methods Appraisal Tool, N/A: not applicable, T2DM: type 2 diabetes mellitus.

We used a standard methodological approach to conduct a systematic review, as used in our previous studies [25,26]. The evidence sourced in the different stages of this review is displayed using a Preferred Reporting Items for Systematic Reviews and Meta-Analysis (PRISMA) flow diagram (Figure 1) [27]. The review aims were structured in a systematic way, using the elements of a clinical question including population, intervention, comparator, and outcome (PICO) [28]. The population (P) included were adults with T2DM and their caregivers, these either being a family member, neighbor, or friend responsible for looking after a person; the intervention (I) was any aspect of online record or service use, the comparator (C) was nonusers of online records or services, and the outcomes (O) were potential impact of online record use or services on the individual (health outcomes), the organization (integration into services), or service technology (current practice information technology [IT] frameworks).

### Search Strategy

Generic and disease-specific searches were developed and run across 9 bibliographic databases focusing on online access to CMR and services from 2004 to October 2016. To ensure evidence was as relevant and up-to-date as possible searches were repeated across databases (EBSCO platform) at the end of the review period. The following databases were searched: MEDLINE, Embase, Cumulative Index to Nursing and Allied Health Literature (CINAHL), PsycINFO, Cochrane database, Cochrane Effective Practice and Organization of Care Group (EPOC), Database of Abstracts of Reviews of Effects (DARE), and the King’s Fund. A search for unpublished material was conducted using the database OpenGrey. Search strings were tailored to each database according to either Medical Subject Heading (MeSH) or index terms and keywords in the title or abstract. Boolean search functions were used (“AND,” “OR,” and “NOT”). An example MEDLINE search string can be seen in Multimedia Appendix 1.

### Inclusion and Exclusion Criteria

Comprehensive inclusion and exclusion criteria were applied to the study, as outlined below.

The inclusion criteria for the found studies were as follows:

- Research focusing on patients and caregivers who have online access to CMRs and online services (which may also include disease-specific portals) via their primary care provider
- Research focusing on patients with T2DM
- Adult patients and their caregiver aged 18 years and over
- All study designs including observational and experimental studies, systematic reviews, and pilot studies which report data.
- Within the date range of 2004-2016

The exclusion criteria for the found studies were as follows:

- When online access to CMR was used by health care staff or researchers only with no patient access
- Studies focusing on the delivery of general health information or education only (information giving) with no online access to CMR by patients
- Studies focusing on the deployment or implementation of new CMR systems in primary care
- Online access to CMR by health care organizations which use data for quality monitoring purposes (ie, Quality and Outcomes Framework [5] only, and do not include any form of patient or carer online access
- A translated copy of article was unavailable
- Research protocols, editorials, or commentary articles were excluded

### Screening

The total number of papers identified was 9970, and of these 3971 were duplicate articles. Over six thousand (6011) titles or abstracts were screened by three authors (FM, MR, and NSAH) for articles matching the inclusion and exclusion criteria. After this process, 92 papers remained for inclusion in the review. These papers were subject to full-text review to see if they entirely fulfilled the inclusion criteria. Any disagreement regarding possible inclusion was resolved by discussing the full-text versions. After full-text review, 28 articles were retained for in-depth analysis. The reference lists of these selected articles were also hand searched for other relevant papers matching the eligibility criteria. Search results and the decisions made regarding inclusion or exclusion of each study were stored using Endnote (v7.4).

#### Data Extraction

A data extraction tool (DEF) was designed by the team to extract relevant information across studies, using Excel. The DEF was initially based on previous designs developed by the first author. The extracted data included the study aims, objectives, population, country of origin, study design, outcomes measures and comparators, methods of analysis used, findings, and study implications. Where possible, all relevant statistical information was also extracted. Data extraction was undertaken independently by two authors (MR and NSH) and checked by FM to ensure consistency and reliability of data being extracted.

#### Quality Appraisal

Data quality was appraised using the Mixed Methods Appraisal Tool (MMAT), an instrument designed to assess the quality of qualitative, quantitative and mixed methods articles [29,30]. The MMAT has five domains each linked to a specific study design; with each domain containing 4 questions. The MMAT has scaled scoring (ie, 25%, 50%, 75%, and 100%). Each article was appraised independently by an author team member, and disagreements were resolved during team meetings. No articles were excluded on the basis of their MMAT score, but more emphasis is placed on articles weighted at 50% or above. Individual scores are presented in the evidence tables. The interrater reliability of the MMAT score is 0.94 [29]. Two raters appraised each study as above. A final total of 28 articles remained and were subject to full data extraction.

#### Data Analysis

Thematic analysis was used to identify themes from the evidence. The analysis was guided by the framework offered by Mayring [31]. This method was chosen as it is sensitive to the diverse type of evidence under study, and the large evidence base. A systematic approach was taken throughout, including the analysis in order to minimize any lack of transparency regarding process or analysis decisions. The heterogeneity of the outcomes across the studies made meta-analysis of results impossible. Where necessary, relevant statistical information is provided for each paper; however, this data is not brought together as trial data were not sufficiently homogeneous in terms of primary outcome to provide a meaningful summary.

## Results

### Study Characteristics

Full data extraction, appraisal, and analysis was conducted on the 28 studies. The majority of the papers originated from the USA (21/28) [32-52], 6 studies were from Europe [53-58], and 1 was Australian [59]. The range of international evidence suggests the international significance of the topic area.

There were a variety of study designs, though the majority employed quantitative methods, using surveys (n=10) [33,35,39,40,45,47,51,54-56] or randomized controlled trials (RCTs, n=5) [34,37,49,52,53]. Several qualitative studies used focus groups and interviews (n=5) [32,36,46,57,59]. Other studies included longitudinal cohort studies (n=3) [38,48,50] and audits (n=3) [41,42,43]. Only one study used a quasi-experimental in design (single interrupted time series) (n=1) [44] and one interpretive review (n=1) [58]. For further information, see Multimedia Appendix 2.

We identified five themes from the studies. These were: (1) disparities in use, (2) improved health outcomes, (3) enhancing self-management support, (4) accessibility, and (5) technical challenges, barriers to use, and system features.

### Disparities in Use

We found disparities based on age, level of deprivation, educational status, ethnicity, and differences in people with more comorbidities. There was greater uptake by those participants with higher income, those who reside in more affluent areas, or those with private insurance.

When considering the age of the participants, users with online access to CMRs and services tended to be younger (59 vs 62 years; *P*<.01) [43,54] or in the 50 to 65 years age band [41,56]. One RCT, which explored the use of an e-journal service, reported little difference in the age of enrollees and nonenrollees (48.9 vs 46.7 years; *P*<.001) [40].

Some studies found that online CMR users had a higher mean annual income (US $53,000 vs US $47,500; *P*<.01) [43], they were said to have higher paid jobs [54] and reside in affluent neighborhoods [41]. In contrast, an RCT that explored the use of an e-journal service, reported little difference in the median income between enrollees and nonenrollees (US $54,617 vs US $52,012; *P*<.001) [40]. Insurance status also influenced online service use, with greater uptake of e-journal use in commercially insured users than those privately insured (84.7% vs 74.7%; *P*<.0001) [40].

Online access to CMR and services was generally reported to be greatest for younger males [41,50,56]. One study suggested women over the age of 65 years were less likely to access services compared to men, who were reported to be more familiar with the internet through employment [41] but one RCT reported little difference in previsit e-journal use by gender at enrolment [40].

Patients who use, or request a log-in, for online CMR access and services were also likely to have a higher level of educational attainment [43,55]. Patients without a university degree (compared to college graduates; odds ratio [OR] 2.3, 95% CI 1.9-2.7) were less likely to log on to online CMRs or services [39].

People with T2DM and with multiple comorbidities and polypharmacy were perceived to have greater diabetes-related stress. These patients were more likely to request access to their CMRs [56]. Additionally, a later survey found greater use of a Web-based portal (related to medicated T2DM patients) by patients experiencing more hypo- and hyperglycemic episodes [54]. A retrospective evaluation study also found the use of shared medical records was greater in patients with higher levels of clinical morbidity [41]. Compared to moderate or lower morbidity, those with high clinical morbidity had a 30% higher rate of ongoing use (rate ratio 1.30, 95% CI 1.16-1.45; *P*<.001); and individuals with very high morbidity had a 21% higher use (rate ratio 1.21, 95% CI 1.07-1.37; *P*=.003). Initial CMR use was also more likely within 3 months of an increase in morbidity (hazard ratio 1.61, 95% CI 1.28-2.01) [41].

There were large differences in the use and uptake of secure messaging (SM) services by different ethnic groups. Black or Hispanic patient groups were less likely to register and use patient online services [38,42]. Similarly, significant differences were found between ethnic minority groups (87.1%) compared to Caucasian (users 69.8%; *P*<.001) in completing a previsit electronic journal (e-journal) about their T2DM targets [40]. African-American and Latino patients were also found to have had higher odds of never logging on to a patient portal (OR 2.6, 95% CI 2.3-2.9 and OR 2.3, 95% CI 1.0-2.6) [51]. Black minority groups were also the least likely to use online services (OR 0.25, 95% CI 0.10-0.63) and the internet [45]. Patients who accessed and used CMR and services were, therefore, likely to be Caucasian (84 users vs 66 nonusers, *P*<0.01) compared to African-American (11 users vs 28 nonusers), and other minority groups [43]. However, in contrast, another study found T2DM patients were more likely to place a positive value on online services if they were male (OR 5.8, 95% CI 0.7-48.9), were from an ethnic group (OR 2.1, 95% CI 0.3-17.6) or had been diagnosed with diabetes within the last 5 years (OR 6.0, 95% CI 0.7-49.8) [33].

A cross-sectional survey also found that ethnicity was a significant predictor of shared medical record (SMR) use. Black (34%, 36/107; OR 0.18, 95% CI 0.11-0.30) and Asian (37%, 35/96; OR 0.40, 95% CI 0.20-0.77) T2DM patients were less likely than Caucasian patients to use SMRs (62%, 265/426; *P*<.01) [45].

Health literacy was also found to play a significant role in the use and uptake of online access to CMR and services. A survey of 14,102 T2DM patients reported that those with limited health literacy were less likely to access a portal than those with adequate health literacy [39]. Of the respondents with limited health literacy, 40% (5671) had higher odds of never signing on to a portal (OR 1.7, 95% CI 1.4-1.9) compared with those who were health literate [39].

Frequency and intensity of CMR access and services were also found to be associated with better diabetes knowledge [54]. Frequency and intensity of service use, such as portal access, could also be associated with different types of health users, for example active or nonactive users [50].

### Improved Health Outcomes

There was a positive association between the use of online access to CMR and services and improved glycemic control [35,37,43,47,48] and general health care management [46]. However, results for blood pressure (BP) were uncertain with some studies reporting improvements in BP outcomes [34,42,43] and other studies reporting either no change in BP outcomes [37], limited change of BP results over time [53] or there were too few patients within the study to provide a meaningful comparison of BP risk [52].

Frequent use of SM between the physician and T2DM patients allowed medication regimes to be optimized more quickly between in-person visits and was associated with improved glycemic control. HbA_1c_ levels (7%) were 36% higher in the SM user group (with 12 or more threads of correspondence) compared to non-SM user groups (relative risk [RR] 1.36, 95% CI 1.16-1.58) when compared with nonmessaging group [35]. A retrospective longitudinal study to determine the extent to which SM is associated with better glycemic control, found that frequent use of SM in the first year was of use is likely to achieve glycemic control (HbA_1c_< 7% and <8%; *P*<.05) [48].

Two further studies found that using Web-based CMR was effective in improving diabetes management [37,43]. A pilot RCT found that HbA_1c_ declined by 0.7%, (*P*=.01; 95% CI 0.2-1.3) an average of 8.2% (7/83) among intervention patients compared to 7.9%, (6/83) with usual care (UC) [37]. However, there was no difference in secondary outcome measures: systolic, diastolic blood pressure, and cholesterol levels between pilot intervention and control groups [37]. Similarly, a retrospective audit of HbA_1c_ levels was 0.29% lower (95% CI –0.35 to –0.23; *P*<.01) after 10 days, compared to nonusers [43].

An RCT comparing clinical outcomes of patients who used a home telemedicine unit (including SM, access to medical record data) to those who receive UC found that intervention group hemoglobin improved compared to UC (0.18%; *P*=.006). Mean systolic and diastolic blood pressure level decreased in the intervention group from 142/71mm Hg to 137/68 mm Hg. The net adjusted reduction for systolic was 3.4 mm Hg (*P*=.001) and for diastolic 1.9 mm Hg (*P*<.001) [34].

Online services such as SM and electronic health reminder letters sent via CMRs also resulted in modest improvements in the management of diabetes care. Greater self-reported use of SM to manage medical appointments were significantly associated with better glycemic control (*P*=−0.29; *P*=0.04) [46]. Automatic electronic health reminder letters (sent via CMR) also showed modest improvement in some diabetes measures, but not all [47]. At the end of 12 months, a CMR letter was effective in achieving compliance targets for testing for HbA_1c_ and low-density lipoprotein (LDL; or 1.24, *P*=.005; or 1.35, *P*=0.03; or 1.48, *P*<.001, respectively). However, these improvements were not sustained with postintervention findings indicating a decline in LDL levels in the following 12 months (0.76, *P*=.003) and in the composite endpoint (or 0.78, *P*=.005) [47]. As such, although the proportion of HbA_1c_ checks improved over a 12-month period, there was an overall gradual decline in achieving an HbA_1c_<7.0% at each time point [47]. Further evidence suggests a decline in effectiveness over time. Although results from an RCT showed an initial significant decline in HbA_1c_ (0.2%) (*P*=.029) systolic (*P*=.036) and diastolic BP (*P*=.035); there were minimal differences between the intervention and control group for these outcomes at 6 months [53].

A study to determine whether physicians who communicate with their patients using (SM and telephone calls) provide better care for patients, found the use of SM within Black or Hispanic groups were associated with improved outcome scores in HbA_1c_, cholesterol and blood pressure (*P*<.01) [42].

### Enhancing Self-Management Support

Self-management support interventions included in CMR access and services facilitated shared management [36,52,57], patients sense of preparedness [40,46,52], and communication with their health care providers [42,46], including contact outside of conventional working hours [32,36].

Record access was initially reported to improve T2DM shared management and decision-making (DM) between physicians and patients [52,57]. This was reported to result in patients’ greater sense of empowerment [52]. A qualitative study showed that self-management of patients’ symptoms also improved with online services, such as access to a diabetes-specific portal [33]. The least-valued function of online services was an electronic information board for patients to share and discuss and answer questions in real time (11/21). In a later focus group, study participants felt more in control of symptoms, valued opportunities to view results, and manage their own medication lists. These patients also received health reminders to monitor personal lifestyle goals in order to remain well [36].

Patients who had online access to CMRs and services were also found to be more prepared for upcoming appointments and were more likely to have medication reviews [46,52]. An RCT found online access to CMR enabled them to forward plan for upcoming appointments ensuring adjustments to treatment regimens (53%, n=82 vs 15%, n=41; *P*<.001) when compared to a control group [52]. Another RCT post-intervention survey to measure satisfaction of an e-journal also found that 55.8% (450/806) of patients were better prepared for doctor visits and 58.0% (467/806) providers held more accurate information [40]. Ease of access to consultation information from home (75.5%, 312/413), and opportunities to monitor disease and treatments (42.5%, 132/413) contributed to patients’ motivation for requesting a CMR login to monitor their diabetes and treatment [54]. Ease of record access and attitudes towards record ownership were also proxies of service quality [57]

Online access to CMRs and services was also found to improve communication with physicians [42,46] as patients were more satisfied when they could view records, request prescription refills, and have personal control over appointment times [36]. A study describing the experience of patients with a chronic medical condition found they valued online services to communicate with physicians’, in comparison to traditional office visits or telephone conversations [32]. Patients also valued seeing results of medical tests online and to track their health status, a need that was previously unmet. Patients felt more secure about managing diabetes symptoms and engaged positively with information provided, especially when the nurse practitioner answered their queries in a timely and consistent manner [32]. Timelines of response was important as users were frustrated when tests results were not released, and messages were not answered [36].

Accessing information outside of normal clinical times was also seen as important [46]. Opportunities for “virtual engagement” outside office hours were reported to potentially reduce demand on providers’ time and encourage self-efficacy. Similarly, 62% (13/21) of patients rated SM as a useful way to communicate with community health care teams and services to manage diabetes care [33].

### Accessibility: Primarily Using Messaging

SM for T2DM patients via CMRs was associated with higher health care utilization, both in terms of outpatient visits [35] and emergency and primary care contacts [36,44]. However, there were no significant changes reported in the number of patient visits or telephone calls received in primary care; from the implementation of a secure communication system [36] and consultation length was largely unaffected [59].

A cross-sectional study found frequent use of CMR messaging was associated with a higher rate of outpatient visits (RR 1.39, 95% CI 1.26-1.53) and suggested an increase of 3-4 additional visits beyond the normal baseline rate of 9 visits per year [35]. Similarly, a study to test whether SM was associated with increased health care utilization and costs found that as the number primary care visits declined, the level of primary care contact actually increased; largely from the use of SM. This single interrupted time series study to evaluate a new initiative (including SM) found emergency visits increased by 9% annually by full implementation. Annual emergency costs also rose by 13% [44].

An earlier interview study which explored the challenges of implementing a secure eHealth software tool (electronic communication system) found no significant change in the number of patient visits or telephone calls received in the office (preintervention, n=21 and postintervention n=18). However, the frequency of CMR and health reminders views increased; as did SM [36].

A feasibility study to explore controlled online access to CMR between general practitioners and patients using a uniquely tailored USB stick (with patient identifier technology) found minimal impact regarding consultation length [59]. However, this system promoted the accuracy of records by patients being able to view their records and report incorrect entries in their medical records [59].

Finally, a pilot RCT using a shared CMR, found care managers reportedly spending 4 hours per week updating care plans and communicating with patients over the Web; thereby potentially lengthening the working day for some professional groups in primary care [37].

### Technical Challenges, Barriers to Use, and System Features

Technical problems with online access to CMR frustrated both patients and providers alike. The consequences were feelings of “disillusionment” with the system and a sense of being “cut off” [32]. Other technical challenges involved lost or unknown passwords and problems with the technical aspects of portals [36]. Other barriers to CMR access were based on expectations as to how online access should work [43] or being unaware of an online portals existence (72.4%, 549/758) [55]. Previous negative experiences and preconceived beliefs or rules about SM were also perceived to be barriers to use [46].

A qualitative study that described the experiences of patients’ use of a disease management program (including CMR access and services) found several recurring themes which may impact on the design and use of Web-based tools for T2DM patient groups [32]. Participants expressed how much they appreciated support in managing nonacute concerns and valuing individual communication at convenient times [32]. Patients desire for individual communication could also potentially be important for patients at specific time points, such as for the newly diagnosed. Being able to upload information about blood glucose with a nurse practitioner also provided participants with a “virtual presence.” Access to real-time health information and timely feedback on medical tests reduced individual worries, which ultimately facilitated better symptom management [32].

Table 1 shows the review article by study design and research focus. Table 2 reports findings by their respective themes. Multimedia Appendices 3 and 4 present a detailed copy of the evidence tables, outlining key points across all references.

### Quality Appraisal Findings

All original studies were subject to MMAT assessment (n=27). The mean MMAT score of included studies was 72% (SD 16.7); indicating moderate to good study quality. Of the 27 included studies, 3 studies were rated as 100% [34,43,49], 19 were rated as 75% [35-37,39-47,50-54,56,57,59], 4 studies were rated as 50% [32,38,48,55], and only 1 study was rated as 25% [33]. See Multimedia Appendix 5, the MMAT Assessment Table for further information.

The majority of the included studies were of moderate to good quality. However, key information relating to outcome measures and comparator groups was occasionally incompletely reported, and some studies lacked detail regarding the description (or processes) of data analysis [33,38,48,56]. MMAT appraisal is useful moving forward as it provides a basis through which to ensure key information is considered at all stages of future research design and reporting.

**Table 1:** Study design, research focus and Mixed Methods Appraisal Tool (MMAT) score of included studies.

Reference	MMAT score	Study design	Study or intervention aim
Ralston et al 2004 [32]	50	Qualitative study using semistructured interviews	To explore the experiences of diabetes management with CMRs^a^ use
Hess et al 2006 [33]	25	Survey and focus group follow up interviews	To evaluate a CMR portal with customized portal features
Shea et al 2006 [34]	100	RCT^b^	To evaluate impact of home telemedicine unit to usual care, on clinical outcomes
Harris et al 2009 [35]	75	Cross-sectional survey	To determine if CMR use is linked to higher quality of care and lower outpatient utilization
Hess et al 2007 [36]	75	Focus groups pre- and postimplementation	To assess patient reaction and challenges with eHealth technology
Ralston et al 2009 [37]	75	Pilot RCT	To test Web-based care management of glycemic control using CMRs
Roblin et al 2009 [38]	50	Longitudinal cohort survey and clustered randomized design	To assess racial preference for registering with a Kaiser Permanente CMR system
Sarkar et al 2010 [39]	75	Survey	Compare use of portal for English-speaking patients versus patients with limited health literacy
Wald et al 2010 [40]	75	RCT-survey	To describe patients experiences of previsit e-Journal use
Weppner et al 2010 [41]	75	Retrospective cohort study	To evaluate the use of SMR^c^ between older patients and provider
Bredfeldt et al 2011 [42]	75	Retrospective study	To determine the relationship between effectiveness SM^d^ or phone calls and Diabetes Recognition Program scores
Tenforde et al 2011 [43]	100	Retrospective audit	To measure the association of CMR use per days and diabetes quality measures
Grembowski et al 2012 [44]	75	Single interrupted time series-design	To examine whether a Group Health Co-operative changed utilization and cost of care
Lyles et al 2012 [45]	75	Cross-sectional survey	To assess the relationship between race or ethnicity and CMR use
Wade-Vuturo 2013 [46]	75	Mixed methods plus focus groups and survey	To explore how adults with T2DM^e^ use a patient portal, to understand nonusers perspectives; and the relationship between SM and glycemic control
Berryman et al 2013 [47]	75	Cross-sectional, practice level study	To evaluate differences in decision making quality metrics at four time points, before and after the introduction of CMR reminders
Harris et al 2013 [48]	50	Retrospective longitudinal cohort plus observational analysis	To determine differences in glycemic control and adherence to HbA_1c_^f^ testing associated with SM
Tang et al 2013 [49]	100	Two-armed RCT. Online questionnaire	To evaluate an online disease management system, compared with usual car
Jones et al 2015 [50]	75	Longitudinal cohort	To describe the types and patterns of portal users in an integrated delivery system
Sarkar et al 2011 [51]	75	Survey	To examine whether social factors influence the use of a patient portal.
Grant et al 2008 [52]	75	RCT	To evaluate the impact of online access to CMR to tailor decision making support and for patient to “develop a plan of care”
Holbrook et al 2009 [53]	75	RCT	To assess the effectiveness of a shared decision support system to improve diabetes care processes & clinical markers
Ronda et al 2015 [54]	75	Survey	To examine patient experiences and use of a Web-portal to access CMR to determine the need for portal redesign
Ronda et al 2014 [55]	50	Cross sectional design/survey	To identify perceived barriers of a Web-based portal to optimize use
Ronda et al 2013 [56]	75	Survey	To examine differences and satisfaction rates of T1DM^g^ and T2DM users or nonusers of a web portal
Fisher et al 2009 [57]	75	Focus groups and telephone interviews	To explore patients’ use of CMR, its benefits, impact, and risks
Jilka et al 2015 [58]	N/A^h^	Interpretative review	To evaluate the impact of a Patient accessible electronic health records for patients to manage personal clinical information
Bomba et al 2004 [59]	75	Feasibility study with field trial and focus groups	To test the feasibility of building a CMR for access using a USB stick (with unique identifier technology). To evaluate USB access

^a^CMR: computerized medical records.

^b^RCT: randomized controlled trial.

^c^SMR: shared medical record.

^d^SM: secure messaging.

^e^T2DM: type 2 diabetes mellitus;

^f^HbA_1c_: glycated hemoglobin.

^g^T1DM: type 1 diabetes mellitus.

^h^N/A: not applicable.

**Table 2:** Themes identified across the included studies.

Reference	Theme 1: Disparities in use	Theme 2: Improved health outcomes	Theme 3: Enhancing self-management support	Theme 4: Accessibility primarily using messaging	Theme 5: Technical challenges, barriers to use, and system features
Ralston et al 2004 [32]			✓		✓
Hess et al 2006 [33]	✓		✓		
Shea et al 2006 [34]		✓			
Harris et al 2009 [35]		✓		✓	
Hess et al 2007 [36]			✓	✓	✓
Ralston et al 2009 [37]		✓		✓	
Roblin et al 2009 [38]	✓				
Sarkar et al 2010 [39]	✓				
Wald et al 2010 [40]	✓		✓		
Weppner et al 2010 [41]	✓				
Bredfeldt et al 2011 [42]	✓	✓	✓		
Tenforde et al 2011 [43]	✓	✓			✓
Grembowski et al 2012 [44]				✓	
Lyles et al 2012 [45]	✓				
Wade-Vuturo 2013 [46]		✓	✓		✓
Berryman et al 2013 [47]		✓			
Harris et al 2013 [48]		✓			
Tang et al 2013 [49]					
Jones et al 2015 [50]	✓				
Sarkar et al 2011 [51]	✓				
Grant et al 2008 [52]		✓	✓		
Holbrook et al 2009 [53]		✓			
Ronda et al 2015 [54]	✓				
Ronda et al 2014 [55]	✓				✓
Ronda et al 2013 [56]	✓				
Fisher et al 2009 [57]			✓		
Jilka et al 2015 [58]					
Bomba et al 2004 [59]				✓	

## Discussion

### Principal Results

Online access appears to be valued by patients with T2DM [32,36,40] but in its current state of development it may widen disparities [39,41,45,55,56]. Males in full-time employment with good IT skills are those most likely to use this service [41]. There appears to be little provision, or development of systems to meet the needs of caregivers; who often provide support outside of working hours.

There are also differences in online access to CMR and services between ethnic groups [38,39,40,42,45]. Black and Asian ethnic groups [45], and Hispanic [42] and African-American male patients [38] were less likely to register and use online services [45], including portals [38,39,40,42]. Only one study suggested gender differences in online access to CMR for African-American patients [38]. Further evidence is needed to explore this area.

Online access to CMR and services is much greater soon after diagnosis, when needs become complex and where changes are needed in medication [32,41,54-56]. Suggesting use could be of benefit to patients at specific time points in their care.

People who take up online services have better glycemic control [35,37,43,48]. However, to date, there is limited evidence of improved outcomes, in either macro- or microvascular complications. Other outcomes such as blood pressure had mixed results either reporting a decline in BP [34,42,43], no change in BP [37], or study limitations which impacted on BP reporting [52,53].

Patients remain concerned about specific aspects of online access to CMR and services including residual worries about how these systems work [43], the rules of engagement in using these systems [46], timeliness of responses from health care professionals [36], and technical failures [32].

### Implications for Future Practice and Research

This review shows disparities between patient groups’ online access to CMR and services to manage diabetes. Greater efforts are needed to make these technologies available to a wider group of patients. This includes across ethnic groups, patients with varying levels of information technology and literacy skills, and age groups. Codesign processes may help identify and meet the needs of patients and caregivers, as their insights may bridge gaps in these new service delivery systems. Further research is needed to understand more about who, why (for what reasons), and when patients use or do not use online access to CMR and services to manage their diabetes.

Online access to CMR and services may need to be tailored to the specific user and condition. This may be particularly important for acute complications for example ketoacidosis. Caregivers may also have different requirements depending on the care recipients specific condition, comorbidities, and wishes about sharing their medical data.

Evidence suggests greater uptake at the time of diagnosis and for a period after, but use does not persist [56]. Further research is needed to explore why use of CMR drops away in the period following initial diagnosis.

Research into physicians and patients views about CMR access in terms of how to provide caregivers appropriate access privileges has not been fully addressed. Whilst physicians are rightly concerned about privacy and confidentiality [58], patients’ concerns focused more specifically on functionality, technical support, and system knowledge [32,36,43,46,55]. It could be that the data needed for monitoring and care in diabetes should have a different level of access, without allowing caregivers comprehensive access to a patients’ record. This might allow sharing of diabetes management with caregivers, with the patient’s consent, without making all their health information available.

Future research should continue to study and address health literacy [38,39,43] and ethnic differences in patients’ access [38,39,40,42,45]. Potential language barriers and lack of explanation of medical terms may also contribute to unequal access [54]. Further research should also be mindful of any unanticipated consequences of online service use in terms of unequal access and use [38].

Online access to CMR and services has also shown to impact on patients’ self-care behaviors which may influence the physician-patient relationship [54,57,60]. It would be interesting to assess in what ways these revised styles of communication impact on service use and/or uptake.

Information technology systems supporting online access to CMR require future development in order to engage and sustain physician and patient use [52]. Tailoring online services to disease-specific conditions may be seen as a valuable resource both in terms of care delivery [33,41,51] and in relation to self-care [33].

Improvements to online access to CMR and services designs may support bundles of care for T2DM management [53] or to improve poorly controlled diabetes [49]. Patient online services could allow targeted approaches to engaging with different population groups with incentives and messages to motivate technology use [50]. However, improving access will be challenging unless there is adequate future funding and training [34].

Integration into primary care business process can be challenging and these include data management [61], communication [42] and costs of implementation and sustainability [44]. Whilst integrating Web-based technology into primary care has been relatively easy [62], health care professionals, may not quickly change their communication patterns [36].

Deployment of online medical records globally is gathering pace [60,63,64]. Within the UK, the importance of online access to CMR and services is growing; as demand for primary care coverage to be available out-of-core working hours (8 am to 6.30 pm Monday to Friday) [65,66] and in response to service needs to support people in the community [67,68].

There are different models of health care delivery and cost, compared to the UK’s National Health Service. Differences may emerge in the use, design, and adoption of online access to CMRs and services. There is a dearth of evidence emerging from the operation of many national CMR systems such as Australia’s “My Health Record System,” Hong Kong’s “Electronic Health record Sharing System” and others in the United States [69].

### Limitations

Like all reviews, evidence has been gathered from various resources from a specific time period. As such there may be several newly published studies that have not been included in this review. Another limitation was the quality of the studies varied (such as poor or incomplete reporting of the study). Findings from the MMAT appraisal indicates possible areas of further development in the design and reporting of studies; particularly in relation to key information such as outcome measures, comparator group data, and description of the data analysis.

All studies reviewed originate from the USA, Australia, and Europe, with little from Africa, Asia, or South America. Limited translation of evidence may have contributed to this lack of evidence. In adhering to the review process, however, every attempt was made to include international evidence which met the inclusion criteria.

### Conclusions

Evidence reported in this review show there are disparities in how different patient groups view, access and use these systems to manage their T2DM. Current users of online CMR access and services tend to be young employed men and they are used less by ethnic minority groups. Uptake is also greater after diagnosis, but then usage falls away, and we are not sure why. Online access is used more where there are complex needs or when medication regimens change. Online access in T2DM is associated with improved glycemic control, but as yet there is no clear evidence of improved outcomes in terms of other complications; such as BP. Concerns remain for patients and physicians about the use and integration of these systems. Further research is ultimately needed into how these systems can meet the needs of wider patient groups. Patient online access to CMR and services to support patients with T2DM are well established internationally and are here to stay.

## Appendix

Multimedia Appendix 1 Medline string.

Multimedia Appendix 2 Study characteristics table.

Multimedia Appendix 3 Evidence table: online computerized medical records and services for patients with type 2 diabetes mellitus.

Multimedia Appendix 4 Continued evidence table: online computerized medical records and services for patients with type 2 diabetes mellitus.

Multimedia Appendix 5 Mixed Methods Appraisal Tool (MMAT).

## Abbreviations

BP: blood pressure
CINAHL: Cumulative Index to Nursing and Allied Health Literature
CMR: computerized medical record
DARE: Database of Abstracts of Reviews of Effects
EPOC: Effective Practice and Organization of Care Group
HbA _1c_: glycated hemoglobin
LDL: low-density lipoprotein
MeSH: Medical Subject Heading
MMAT: Mixed Methods Appraisal Tool
OR: odds ratio
PICO: population, intervention, comparator, and outcome
PRISMA: Preferred Reporting Items for Systematic Reviews and Meta-Analysis
RCT: randomized controlled trial
RR: relative risk
SM: secure messaging
SMR: shared medical record
T1DM: type 1 diabetes mellitus
T2DM: type 2 diabetes mellitus
