# Supplementary material for: The Impact of Patient Online Access to Computerized Medical Records and Services on Type 2 Diabetes: Systematic Review
Source: J Med Internet Res. 2018 Jul 6;20(7):e235. doi: 10.2196/jmir.7858 (PMC6054706; doi:10.2196/jmir.7858)
Supplement: Multimedia Appendix 1 [file jmir_v20i7e235_app1.pdf]

Multimedia Appendix 1: Example Medline Search String

Diabetes Online Access String	
Database: Medline<br>Platform: EBSCO<br>Limits: 2012 to 2016, Human only	
1	(MH "Medical Informatics")
2	“emr*”.ti.ab OR “cmr*”.ti.ab
3	"electronic medical record*".ti.ab OR "electronic health record*".ti.ab
4	“medical record*”.ti.ab
5	(MH "Electronic Health Records")
6	"computeri*ed medical record*".ti.ab OR "computeri*ed health record*".ti.ab
7	(MH "Medical Records Systems, Computerized")
8	(MH "Medical Records")
9	(MH "Health Records, Personal")
10	(MH "Records as Topic")
11	(MH "Access to Information")
12	(MH “Patient Access to Records”)
13	OR/1-12
14	(web* OR internet OR www OR electronic* OR online OR “electronic mail*” OR email* OR e-mail* OR “web mail*” OR webmail* OR “internet mail*” OR messag*).ti.ab
15	(patient OR health OR information OR web OR internet) N5 portal*.ti.ab
16	(“ehealth” OR “e-health”) NOT ((MH "health promotion") OR “health promotion” OR “promotion of health”).ti.ab
17	(e-prescri* OR electronic prescribe* OR e-health) AND (web* OR internet). ab.ti
18	(online OR web* OR internet) N4 (consult* OR service* OR intervention* OR therap* OR treatment* OR counsel*).ti.ab
19	(web* OR internet OR www OR electronic* OR online) N5 (messag* OR communicat* OR transmit* OR transfer* OR send* OR deliver* OR feedback OR letter* OR interactive* OR input* OR forum OR appointment* OR booking* OR referral* OR consult* OR prescri*).ti.ab
20	(MH "Triage") AND (web form* OR web chat* OR web based triage or web)
21	OR/15-20
22	(MH "Diabetes Mellitus, Type 2+")
23	Diabet*.ti.ab
24	(NIDDM or MODY or T2DM or T2D).ti.ab
25	(non$insulin$depend*).ti.ab
26	OR/22-25
27	(MH "General Practice")
28	(MH "General Practitioners")
29	(MH "Family Practice")
30	(MH "Primary Health Care")
31	(MH “ambulatory care”) OR (Ambulatory Care Facilitates”)
32	“primary care”
33	“community-based provider*”.ti.ab
34	OR/27-33
35	13 AND 21 AND 26 AND 34
**Key**<br>Ti– title word<br>Ab – abstract word<br>MH – Main index/ MeSH term	
