# Supplementary material for: The Impact of Patient Online Access to Computerized Medical Records and Services on Type 2 Diabetes: Systematic Review
Source: J Med Internet Res. 2018 Jul 6;20(7):e235. doi: 10.2196/jmir.7858 (PMC6054706; doi:10.2196/jmir.7858)
Supplement: Multimedia Appendix 2 [file jmir_v20i7e235_app2.pdf]

7854-126336-3-RV-FM Appendix 2 20180417.docx

Multimedia Appendix 2: Study Design/ Characteristics

**Study Design**	**Country, Number**	**Author, Year & Reference**
**Audit (Retrospective)**	USA, 3	Bredfeldt et al (2011) [42]<br>Weppner et al (2010) [41]<br>Tenforde et al (2011) [43]
**Cohort studies (retrospective, prospective and longitudinal)**	USA, 3	Jones et al (2015) [50]<br>Roblin et al (2009) [38]<br>Harris et al (2013) [48]
**Quasi-experimental (single interrupted time series-design)**	USA, 1	Grembowski et al (2012) [44]
**Interpretative Review**	UK, 1	Jilka et al (2015) [58]
**Focus groups/ Interviews**	USA, 3	*Wade-Vuturo et al (2013) [46]<br>Hess et al (2007) [36]<br>Ralston et al (2004) [32]
	Australia, 1	Bomba et al (2004) [59]
	UK, 1	Fisher et al (2009) [57]
**Randomised / Control & Cluster Trials (RCT)**	USA, 4	Shea et al (2006) [34]<br>Ralston et al (2009) [37]<br>Tang et al (2013) [49]<br>Grant et al (2008) [52]
	France, 1	Holbrook et al (2009) [53]
**Survey (including cross-sectional/ analysis)**	USA, 7	Wald et al (2010) [40]<br>Sarkar et al (2010) [39]<br>Sarkar et al (2011) [51]<br>Berryman et al (2013) [47]<br>Lyles et al (2012) [45]<br>Harris et al (2009) [35]<br>Hess et al (2006) [33]
	The Netherlands, 3	Ronda et al (2013) [56]<br>Ronda et al (2014) [55]<br>Ronda et al (2015) [54]

*Mixed methods (interviews & survey)
