# Supplementary material for: The Impact of Patient Online Access to Computerized Medical Records and Services on Type 2 Diabetes: Systematic Review
Source: J Med Internet Res. 2018 Jul 6;20(7):e235. doi: 10.2196/jmir.7858 (PMC6054706; doi:10.2196/jmir.7858)
Supplement: Multimedia Appendix 3 [file jmir_v20i7e235_app3.pdf]

Multimedia Appendix 3: Evidence Tables - Online CMR & Services for patients with T2DM

Key terms: CMR (Computerized Medical Records), GP (General Practitioner), PHC (Primary Health Care), Usual Care (UC), Confidence Interval (CI), Focus Groups (FGs), RCT (Randomized Controlled Trial) T2DM (Type 2 Diabetes Miletus), LDL (low-density lipoprotein), HbA_{1c} (glycated hemoglobin),

Reference, Country, MMAT Score	Study Design	Participation / Population (T2DM), Participants & Settings	Study/ Intervention Aim	Instruments Used/ Tools<br><br>Primary, Secondary and Comparator Groups
Ralston et al 2004 [32], USA, 50	Qualitative study using semistructured interviews	T2DM aged 43-65 years n=9/35 adults, PHC	To explore the experiences of diabetes management with CMRs use	Access to CMR emails, blood glucose readings, education site, an interactive online diary for exercise, diet and medication regimes
Hess et al 2006 [33], USA, 25	Survey 5 FGs follow up interviews	DM mean age 53.4 years, n=21/ no total, 3xPHC	To evaluate a CMR portal with customized portal features	Patient portal for preventative health reminders, appointments, medication lists and educational materials
Shea et al 2006 [34], USA, 100	RCT	T2DM Aged ≥ 55 years. n=1665/ 9,597 HbA_{1c}>7.0%, PHC	To evaluate impact of home telemedicine unit to usual care, on clinical outcomes	Home telemedicine unit
Harris et al 2009 [35], USA, 75	Cross-sectional survey	T2DM. Mean ages 58 and 63 years. n=5274/ 15,427, PHC	To determine if CMR use is linked to higher quality of care and lower outpatient utilization	A website for SM, prescription refills, appointment scheduling, access to medical records

Hess et al 2007 [36], USA, 75	5 FGs (n=21) pre-implementation 5 FG (n=18) post-implementation	DM Mean age 54, n=39/no total, 3x PHC	To assess patient reaction and challenges with eHealth technology	eHealth personal record system comprising of CMR information, health reminders and SM to provide shared information with the provider, offering preventive health care reminders, and general health information
Ralston et al 2009 [37], USA, 75	Pilot RCT	T2DM Mean age 59.4 vs 57.9 n=83/ 709 HbA_{1c}<7.0%, PHC	To test Web-based care management of glycemic control using CMR	Access to interactive online CMR diary for recording, BG, medications, exercise diet and SM. Systolic & diastolic BP, total cholesterol levels (TCL)
Roblin et al 2009 [38], USA, 50	Longitudinal cohort survey clustered randomized design.	T2DM Adults > 25 years n=1,777/ 5,309, PHC	To assess racial preference for registering with a Kaiser Permanente CMR system	Online access to CMR for appointments, refill prescription, lab results, complete a health appraisal and health information
Sarkar et al 2010 [39], USA, 75	Survey	T2DM Mean age 52 years n=14,102/40, 735, PHC	Compare use of portal for English-speaking patients versus patients with limited health literacy	Web-portal access for health promotion, lab results, email from physicians, medications refills, medical appointments, Health insurance information
Wald et al 2010, [40], USA, 75	RCT- survey	T2DM Mean ages 43 years n= 3,979/ 21,533, PHC	To describe patients experiences of pre-visit e-Journal use	eJournal linked to CMR via portal. Purpose was to review clinical information in preparation for a face-to-face visit with provider
Weppner et	Retrospective	T2DM	To evaluate the	Shared medical record

al 2010 [41], USA, 75	cohort study	patients aged >65 years n=6185/7,076, PHC	use of SMR between older patients and providers	with a SM service, medication refills requests, personal appointments, viewing test results, after-visit summaries, medical problem lists, allergies, and immunizations
Bredfeldt et al 2011 [42], USA, 75	Retrospective study	DM >18 years n=174 primary care physicians/ Total population not reported, HbA_{1c}>7.5%, PHC	To determine the relationship between effectiveness SM or phone calls and Diabetes Recognition Program scores (DRP)	Use of descriptive statistics and linear regression models to examine the use of SM/phone and DRP scores. Use of a Generalized Estimating Equation model
Tenforde et al2011 [43], USA, 100	Retrospective audit	T2DM Mean age 47 years n=4036 record users/10,746, PHC	To measure the association of CMR use per days and diabetes quality measures	Diabetes quality control measures including; HbA1c, LDL, BP, BMI, hypertensive medications (angiotensin-converting enzyme inhibitors (ACEi)/ angiotensin-receptor blockers (ARB), microalbumin testing, pneumococcal vaccinations, food & eye exam, and smoking status
Grembowski et al 2012 [44], USA, 75	Single interrupted time series-design	T2DM mean n=4971/9871 age 63 HbA_{1c}<7.0%, PHC	To examine whether a Group Health Co-operative (GHC) changed utilization and cost of care	The evaluation of a new service called Access Initiative (AI) comprising of secure email to physicians and access to electronic medical records
Lyles et al 2012 [45],	Cross-sectional	DM >65years n=718/910,	To assess the relationship	CMR allowed viewing after-visits, doctor

USA,<br>75	survey	PHC	between race/ethnicity and CMR use	summaries, medical history and diagnoses, appointments, prescriptions refills and lab results
Wade-Vuturo 2013 [46]<br>USA,<br>75	Mixed methods: focus groups and survey	T2DM<br>Mean age 57 years.<br>Analysis from n=39 FG (users) and n=54 (total) survey respondents/ total study population not reported, PHC	To explore how adults with T2DM use a patient portal, to understand non-users perspectives; and the relationship between SM and glycemic control	A patient portal enabling access to CMR and SM to communicate with providers, manage medical appointments and bills
Berryman et al 2013 [47], USA,<br>75	Cross-sectional, practice level study.	T2DM > 18 years, Mean age 40 years.<br>TP1 n=1020<br>TP2 n=1021<br>TP3 n=1000<br>TP4 n=1025/ total population not reported HbA_{1c}<7.0%, PHC	To evaluate differences in DM quality metrics at four time points (TPs), before and after the introduction of CMR reminders	Automatic CMR reminder letters sent when records showed non-compliance for quality metrics; HBA_{1c}, LDL, and BP
Harris et al 2013 [48], USA,<br>50	Retrospective longitudinal cohort. Observational analysis.	T2DM>18 years n=6301/ 15,438 HbA_{1c}<7.0%, <8.0%, <9.0%, PHC	To determine differences in glycemic control and adherence to HbA_{1c} testing associated with SM	A website for prescription refills, appointment scheduling, computerized medical records and SM access.<br>HbA_{1c}< 7%, HbA_{1c}<8% and HbA_{1c}>9%.
Tang et al 2013 [49], USA,<br>100	Two-armed RCT. Online questionnaire	T2DM aged>18 years n=415/6907	To evaluate an online disease management system,	Wireless home access to a glucometer, diabetes summary status, nutrition &

		HbA_{1c}>7.5%,<br>PHC	compared with usual care	exercise logs, insulin records and online messaging with health team. Other services include access to medication management advice, personalized texts/ video educational sessions. LDL, BP
Jones et al 2015 [50],<br>USA,<br>75	Longitudinal cohort	T2DM comparison of n=3297 users and n=1648 non-users/<br>n= 4945 (total),<br>PHC	To describe the types and patterns of portal users in an integrated delivery system	A web-based portal to enable access to medications, problem lists, preventative health reminders, office visits, medication refills, appointments & requesting referrals, HbA_{1c}, LDL, BP and BMI results
Sarkar et al 2011 [51],<br>USA,<br>75	Survey-validated and use in a previous study.<br>Response rate 62%	T2DM<br>Mean age 52 years.<br>n=5671 (40%) requested portal passwords/<br>n=14,102,<br>HbA_{1c}<7.0%,<br>PHC	To examine whether social factors influence the use of a patient portal	Portal access for lab results, email from physicians, medications refills, medical appointments
Grant et al 2008 [52],<br>USA,<br>75	RCT	T2DM<br>Mean age 46 years<br>n=244/<br>6797, PHC	To evaluate the impact of online access to CMR to tailor DM decision support and for patient to 'develop a plan of care'	DM-specific computer medical record linked directly to primary care physician.<br>Blood pressure and LDL-C control
Holbrook et al 2009 [53],<br>France,	RCT	T2DM<br>Mean age 60.7<br>n=511/1610,	To assess the effectiveness of a shared decision	Shared access to web-based diabetes tracker clinical composite

75		PHC	support system to improve diabetes care processes & clinical markers	scores, QOL scores, continuity of care & usability
Ronda et al 2015 [54], Netherlands, 75	Survey. Response rates 42.8% (n=632/ 1476)	DM<br>Mean age 59.7 yrs.<br>n=1500,<br>PHC	To examine patient experiences and use of a Web-portal to access CMR to determine the need for portal redesign	The Web-portal aimed to enhance shared medical record access including access to test results, problem lists, and treatment goals
Ronda et al 2014 [55], Netherlands, 50	Cross sectional design/ survey. Response rate 66.63%, n=2391	DM<br>Mean age 63.9.<br>n= 2931/ 4500, x62<br>PHC	To identify perceived barriers of a web-based portal to optimize use	Web portal comprising of access to medical records, medical consultation information, test results, problem lists and treatment goals
Ronda et al 2013 [56], Netherlands, 75	Survey. Response rates 66.63%, n=2931	T2DM<br>Mean age 63.9 yrs.<br>n= 1500/ 4500, x62<br>PHC	To examine differences and satisfaction rates of T1DM and T2DM users or non-users of a web portal	Web portal to access CMR through the internet
Fisher et al, 2009 [57], UK,<br>75	FGs and telephone interviews.	DM > 20 years n=43 Total population not reported, PHC	To explore patients use of CMR, its benefits, impact, and risks	A service offering electronic access to full medical records (including details on consultation prescriptions, test results, and vaccinations
Jilka et al 2015 [58], UK<br>MMAT: N/A	Interpretative review (n=10)	Patients with diabetes and hypertension.<br>PHC	To evaluate the impact of a Patient accessible electronic health records for patients to	A systematic review focusing on the impact of electronic health record access from patients and HCPs perspectives

			manage personal clinical information	
Bomba et al 2004 [59], Australia, 75	Feasibility study - field trial. FG interviews to develop a questionnaire	T2DM<br>n=6 GPs<br>n=20 patients. Total population not reported, PHC	To test the feasibility of building a CMR for access using a USB (universal serial bus) stick (with unique identifier technology).<br>To evaluate USB access	UBS technology system to automatically collect data linked to remote CMR
