# Supplementary material for: The Impact of Patient Online Access to Computerized Medical Records and Services on Type 2 Diabetes: Systematic Review
Source: J Med Internet Res. 2018 Jul 6;20(7):e235. doi: 10.2196/jmir.7858 (PMC6054706; doi:10.2196/jmir.7858)
Supplement: Multimedia Appendix 4 [file jmir_v20i7e235_app4.pdf]

Multimedia Appendix 4: Continued Evidence Tables - Online CMR & Services for patients with T2DM

Key terms: CMR (Computerized Medical Records), GP (General Practitioner), PHC (Primary Health Care), Usual Care (UC), Confidence Interval (CI), Focus Groups (FGs), RCT (Randomized Controlled Trial) T2DM (Type 2 Diabetes Miletus), LDL (low-density lipoprotein), HbA_{1c} (glycated hemoglobin),

Reference, Country, MMAT Score	Statistical Test Used	Main Findings (outcome measure findings)	Key Recommendations & Implications
Ralston et al 2004 [32], USA, 50	Qualitative thematic analysis	Patients valued;<br>• Individual concerns being heard when newly diagnosed and with acute concerns.<br>• Virtual presence of NP to answer health queries in a timely and consistent manner Online access to medical tests to track health status and being able to communicate with NPs<br>Least valued;<br>• When NPs did not communicate results, patients felt disappointed<br>• Technical failures & being 'cut off' online	Research needs to focus on how to design future CMR programs for T2DM patients' individual needs
Hess et al 2006 [33], USA, 25	Descriptive statistics	• Most frequent users of the portal were male, from ethnic minority groups and newly diagnosed within 5 years.<br>• Virtual engagement encourages review of results, & management of medication lists	Future work needs to address the usefulness of portals for disease-specific patients

		• Frequent health reminders sent to monitor personal lifestyle goals, to remain well<br>• 62% (13/21) of patients rated SM as a useful way to communicate with health care teams	
Shea et al 2006 [34] USA,<br>100	Descriptive statistics<br>2 tailed test	Adjusted net reductions favoring the intervention were;<br>• HbA_{1c}, 0.18% (*P*=.006), systolic and diastolic blood pressure, 3.4 (*P*=.001) and 1.9 mm Hg (*P*<.001), and LDL cholesterol, 9.5 mg/dL (*P*<.001)	Barriers to use include lack of data systems resources to upload data. Cost and training and technology are influencing factors
Harris et al 2009 [35], USA,<br>75	Multivariate regression analysis	• SM was associated with better glycemic control and increased outpatient utilization<br>• After multivariable analysis the rate of AIC<7% was 36% higher infrequent users of SM (>12 message threads) compared to non-messaging user groups (relative risk [RR] 1.36; 95% CI 1.16–1.58)<br>• Similarly, rates of primary care visits were 32% higher for SM user groups, compared to non-user groups<br>• This results in higher numbers of outpatient visits between frequent users (RR 1.39, 95% CI 1.26–1.53) and non-SM users (RR 0.98, 95% CI 0.95-1.02, n=15,237, *P*<.001)	More research is needed to show links between SM and better medication adherence to improve glycaemic control
Hess et al 2007 [36], USA,<br>75	Grounded theory analyses	• e-Technology improved communication and self-efficacy<br>• SM increased, but no change in the number of patient visits or telephone calls<br>• Increased satisfaction linked with viewing records, request	Future work should focus on understanding barriers to Diabetes self-management

		prescription refills, and control over appointment times<br>• Patients frustrated when messages were unanswered, and with technical software issues	
Ralston et al 2009 [37], USA, 75	Linear regression.	• HbA_{1c} declined by 0.7% (95% CI 0.2–1.3) compared with UC at 12 months<br>• BP and TCL levels did not differ between groups<br>• The decrease in GHb was concordant with telephone follow- up from care manager	Web-based care management has the potential to improve glycemic control in patients with T2DM
Roblin et al 2009 [38] USA, 50*	Descriptive statistics<br><br>SAS version 9.1 and	• Registrants more likely to be Caucasian and African American females (Caucasian 54.8%, n=407, African American 65.3%, n=564 compared to both Caucasian and African American males (Caucasian 45.2%, n=336, African American 34.7%, n=300)<br>• Registration was influenced by levels of education, and less likely for African Americans<br>• Unintended consequences of digital services are widening of disparities due to unequal access and use	Further research needs to identify the causes of racial and ethnic disparities in accessing digital services. Deficits in education, computer literacy, and IT access may widen disparities in health care
Sarkar et al 2010 [39], USA, 75	Descriptive statistics	• 62% (n=6099) reported some limitation in health literacy<br>• 40% (n=5671) respondents completed portal registration<br>• In adjusted analyses, those with limited health literacy had higher odds of never signing on to the patient portal (OR 1.7, 95% CI 1.4-1.9) compared with those who did not report any health literacy limitation	Those most at risk of poor diabetes health outcomes are also at further risk of falling further behind if health systems increasingly rely on patient portal health services

Wald et al 2010 [40], USA, 75	Descriptive statistics	• Minimal differences in age (48.9 vs 46.7 years, *P*<.001), gender (60.2% vs 64.7% female, *P*<.001) and median income (US $54 617 vs US $52 012, *P*<.001) of enrollees and non-users in journal use<br>• Larger differences observed by ethnicity (87.1% vs 69.8% Caucasian, *P*<.001) and insurance (84.7% vs 74.7% privately insured, *P*<.001)<br>• Patients who prepared for visits were more satisfied, because providers' information was more accurate about them, which improved communication with physician	Further work needed to improve journal integration into physicians practice workflow, and documentation
Weppner et al 2010 [41], USA, 75	Cox proportional hazard analysis	• SMR use was associated with younger age, male sex, higher socioeconomic status and overall morbidity levels<br>• Initial SMR use was more likely within 3 months of an increase in morbidity (hazard ratio [HR] 1.61, 95% CI 1.28–2.01) and within 1 month of changing to a PCP with higher use (HR 3.02, 1.66–5.51)<br>• Web-based SMRs may provide features that are useful to patients with increased morbidity. Endorsement by providers is necessary	Providing features that are useful to patients with chronic disease and higher morbidity may be important to promote adoption<br><br>Need to evaluate longer-term effects of SMRs on disease self-management
Bredfeldt et al 2011 [42], USA, 75	Descriptive statistics	• Physicians who communicate with T2DM via SM significantly improves Diabetes Recognition Program scores.<br>• The use of SM and phone by Black or Hispanic groups was associated with improvements	Research is needed to understand how different types of SM communication methods impact different populations groups

		in outcomes scores (HbA_{1c}, Cholesterol, BP) $P< 0.01$)	
Tenforde et al 2011 [43], USA, 100	Multivariable logistic regression	• PHR users were younger, had higher incomes, educational attainment, were more likely to identify as Caucasian, and had better unadjusted and adjusted diabetes quality measure profiles<br>• Adjusted odds ratio of HbA_{1c} testing was 2.06 (P<.01) and most recent HbA_{1c} was 0.29% lower (P <.01)<br>• PHR users had lower systolic and diastolic BP values than non-users (P<0.01)	Demographic variables related to age, income health literacy, cultural differences and language barriers need addressing. PHR software programs should be designed with patients
Grembowski et al 2012 [44], USA, 75	Linear regression models	• Implementation of the Access Initiative (AI) service increased utilization costs and the annual rate of change<br>• Emergency visits increased by 9% annually<br>• Emergency costs rose by 13%, attributed to comorbidity of T2DM	Balancing trade-offs between service costs and quality may have unintended consequences, such as reduced efficiency
Lyles et al 2012 [45], USA, 75	Chi-squared X^2 and 2 sided t tests	• There was no association between provider encouragement and shared medical record use by ethnicity<br>• However, in fully adjusted models, black participants [odds ratio (OR) 0.18, 95% CI 0.11-0.30] and Asian participants (OR 0.40, 95% CI 0.20-0.77) were significantly less likely than Caucasian participants to use the CMR<br>• Analysis of those with limited internet use found access to SMR remained the same for black minority groups (OR, 0.25, 95% CI 0.10-0.63)	Future work should study racial or ethnic differences in patients' access and use of CMR

Wade-Vuturo 2013 [46], USA, 75	Fisher exact & Mann-Whitney U Spearman's correlation coefficient	• Self-reported satisfaction, efficiency and better preparation for face-to-face visits, and access outside office hours<br>• Use of SM to manage a medical appointment was significantly associated with patients glycemic control (*P*=−.29, *P*=.04)<br>• Providing up-to-date medication and problem lists in the electronic health record (EHR), avoiding unnecessary office visits<br>• Opportunity to communicate directly with the provider	In future, it is critical that providers have protected time to devote to patients' messages.
Berryman et al 2013 [47], USA, 75	Descriptive statistics	• Automatic CMR reminder letters showed modest improvement in some DM measures.<br>• HbA_{1c} checks improved over a 12-month period.<br>• A gradual decline in HbA_{1c} <7.0% at each time point.	The implications of this study are that automatic CMR for DM patients not meeting HbA_{1c}, LDL or PB goals improves overall process outcome measures related to LDL and HbA_{1c} checks
Harris et al 2013 [48], USA, 50	Log-Linear regression models	• Greater use of insulin and better glycemic control were associated with greater SM use<br>• Adjusted rate of good glycemic control was higher in relation to higher level of SM in the first year (HbA_{1c}< 7% and < 8% P<.05)	SM may modify testing frequency by increasing provider accessibility. Several variables affect pathways for SM, therefore exploring patients' views may enhance patient engagement.
Tang et al 2013 [49], USA, 100	Chi-squared X^2 and *t*-tests	• Intensification of diabetes treatment (such as an additional treatment or increased dosage of an existing medication) increased in the intervention group (563 vs	A nurse-led multidisciplinary team can manage a population of diabetic patients in an online

		401, *P*=.001) compared with the usual care group.<br>• There were no significant difference in the total number of diabetes-related physician visits between the intervention and usual care groups (2.4 (2.0) vs 2.3 (1.9); *P*=0.46)	disease management program
Jones et al 2015 [50], USA, 75	Factor & cluster analysis	• Typologies of portal user types of eHealth users such as eDabblers, infrequent intense users, electronic messengers, appointment preparers, lab trackers, biometric monitors, proxy moms, and record updaters<br>• Frequency and intensity of portal use could discriminate various types of health users	Predictive capabilities may engage different population groups with incentives & messages that can motivate eHealth use and develop new types of software technologies
Sarkar et al 2011 [51], USA, 75	Multivariate logistic regression	• African American participants and Latino participants had higher odds of never logging on (OR 2.6, 95% CI 2.3-2.9); OR 2.3 (95% CI 1.9-2.6), as did those without an educational degree (OR compared to college graduates, 2.3 (95% CI 1.9-2.7), compared with non-Hispanic Caucasian participants.	The internet has potential to use visuals, spoken, or multilingual techniques to meet the needs of disadvantaged groups
Grant et al 2008 [52], USA, 75	SAS version 9.0 and Chi-squared X^2 test	• More patients in the intervention arm had their DM treatment regimens adjusted, and have medication reviews (53%, n=82) vs 15%, n=41; *P*<.001) compared with active controls	Web-based patient portals require significant redesign to engage physicians and patients in non-visit-based care
Holbrook et al 2009 [53], France, 75	Chi-squared X^2 and 2 sided *t* tests	• HbA_{1c} declined only 0.2% more in the intervention group (n=253), compared to the control group (n=258, *P*=.03)	More research into eHealth technology and through supportive funding

		• Better patient satisfaction reported in the intervention group when compared to UC	
Ronda et al 2015 [54], USA, 75	Data analyzed using SPSS v20, Chi-squared X^2, unpaired *t* tests and logistic regression	• Users were younger with a paid job, had better diabetes knowledge, used insulin and experienced more hypo/hyperglycemic episodes<br>• Ease of access to consultation information from home (75.5%, 312/413), and an opportunity to monitor disease and treatments (42.5%, ,132/413) motivated self-care.	Portal designs need to help patients to make appropriate lifestyle changes. The language used on portals needs to be simplified and less jargonistic Medical terms require explanation and abbreviations
Ronda et al 2014 [55], Netherlands, 50	Chi-square X^2 and Mann-Whitney *U* tests	• T2DM patients with complex symptoms, on 5 or more medications, perceived more diabetes-related distress but had better knowledge of their disease and more likely to request a log-in<br>• Age, gender, educational attainment and work status played an important role in requesting a log-in	Web-based portals have the possibility of reducing visits to out-patient clinics without compromising the quality of care
Ronda et al 2013 [56], Netherlands, 75	Chi-squared X^2 & Mann-Whitney *U* tests. Data analyzed using SPSS v20	• Regular users (75.5%, 312/413) motivated to access results from home. Only 32% (132/413) used the opportunity to monitor disease and treatments, and 17.9% (74/413) accessed physician recommendations. Just 0.5 % (2/413) were dissatisfied with current care	Better strategies have to be found to inform patients about Web-based portals, how to request a login and advise on the long-term benefits a portal may offer
Fisher et al 2009 [57], UK, 75	NVIVO v2 - Content Analysis	• Record access improved shared management and decision making between doctor and patient<br>• Empowerment through self-care or management improved communication, enhanced	Future studies need to focus on the measurement of these outcomes once electronic access becomes well-established

		patient-doctor relationships, and adherence to treatment<br>• Attitudes to quality of care delivery focused on speed of access, continuity, and ownership of health records	
Jilka et al 2015 [58], UK, MMAT: N/A	Interpretative Review	• There is currently insufficient evidence about the effect of patient accessible electronic health records (PAEHRs) on health outcomes related to; patients' safety, usefulness, satisfaction and self-efficacy across patients and HCPs.<br>• Only 50% of studies (5/10) showed positive changes in online access to CMRs<br>• Patients believe that access to CMRs increases perception of control but viewing results can create anxiety<br>• Nurses more likely than physicians to gain time efficiencies with CMRs use<br>• Physicians' main concern is online security	Current research is too targeted within certain clinical groups. Research should address understanding of how access CMRs can bridge the gap between patients and HCPs, using up to date technologies
Bomba et al 2004 [59], Australia, 75	Descriptive statistics and content analysis	• A unique USB (Universal Serial Bus) stick which allows patients and GPs controlled access to CMR, but does not reduce patient consultations times<br>• The USB system promotes patient empowerment, reducing the possibility of GP as gatekeeper role of patient	USB system is seen as an acceptable innovation which could be rolled out to the wider community
