# Supplementary material for: The Impact of Patient Online Access to Computerized Medical Records and Services on Type 2 Diabetes: Systematic Review
Source: J Med Internet Res. 2018 Jul 6;20(7):e235. doi: 10.2196/jmir.7858 (PMC6054706; doi:10.2196/jmir.7858)
Supplement: Multimedia Appendix 5 [file jmir_v20i7e235_app5.pdf]

7858-126336-3-RV – Multimedia Appendix 3: MMAT Quality Appraisal Table

Author, Year, Reference	Research question		Qualitative				Quantitative RCT				Quantitative Non-RCT				Quantitative descriptive				Mixed methods			MMAT %
	Clear RQ?	Data match to RQ?	1.1	1.2	1.3	1.4	2.1	2.2	2.3	2.4	3.1	3.2	3.3	3.4	4.1	4.2	4.3	4.4	5.1	5.2	5.3	
Berryman et al, 2013 [47]	Y	Y													Y	Y	Y	C				75%
Bomba et al, 2004 [59]	Y	Y													Y	Y	Y	C				75%
Bredfeldt et al, 2011 [42]	Y	Y									Y	N	Y	Y								75%
Fisher et al,2009 [57]	N	C	Y	Y	Y	C																75%
Grant et al, 2008 [52]	Y	Y					Y	N	N	N												75%
Grembowski et al, 2012 [44]	Y	Y													Y	N	Y	Y				75%
Harris et al, 2009 [35]	Y	Y									C	Y	Y	Y								75%
Harris et al, 2013 [48]	Y	Y									C	Y	C	Y								50%
Hess et al, 2006 [33]	N	N	C	C	Y	N																25%
Hess et al, 2007 [36]	N	C	Y	Y	N	Y																75%
Holbrook et al, 2009 [53]	Y	Y					Y	Y	Y	C												75%
Jilka et al 2015 [58]	-	-	-	-	-	-	-	-	-	-	-	-	-	-	-	-	-	-	-	-	-	-
Jones et al, 2015 [50]	Y	N									Y	C	Y	Y								75%
Lyles et al, 2012 [45]	N	C									Y	Y	Y	N								75%
Ralston et al, 2004 [32]	Y	Y	Y	Y	N	C																50%
Ralston et al, 2009 [37]	Y	Y					Y	Y	C	Y												75%
Roblin et al, 2009 [38]	Y	Y									N	N	Y	Y								50%
Ronda et al, 2013 [56]	Y	Y													Y	N	Y	Y				75%
Ronda et al, 2014 [55]	Y	Y													Y	C	N	Y				50%
Ronda et al, 2015 [54]	Y	Y													Y	Y	Y	N				75%
Sarkar et al, 2010 [39]	Y	Y									N	Y	Y	Y								75%
Sarkar et al, 2011 [51]	Y	Y													Y	N	Y	Y				75%
Shea et al, 2006 [34]	Y	Y					Y	Y	Y	Y												100%

Tang et al, 2013 [49]	Y	Y					Y	Y	Y	Y												100%
Tenforde et al, 2011 [43]	Y	Y									Y	Y	Y	Y								100%
Wade-Vuturo, 2013 [46]	Y	Y																	Y	Y	C	75%
Wald et al, 2010 [40]	Y	Y					Y	N	Y	Y												75%
Weppner et al, 2010 [41]	Y	Y									Y	Y	Y	N								75%

MMAT Key: Y: Yes; N: No; C: Can’t tell
